# Supplementary material for: Proton range monitoring using 13N peak for proton therapy applications
Source: PLoS One. 2022 Feb 15;17(2):e0263521. doi: 10.1371/journal.pone.0263521 (PMC8846528; doi:10.1371/journal.pone.0263521)
Supplement: S1 Appendix — (DOCX) [file pone.0263521.s001.docx]

**Appendix A**

The PeakCalib is a standalone open-source computer program that performs linear and non-linear cubic spline interpolation of the obtained data from PHITS Monte Carlo package. PeakCalib software and source code can be downloaded from: <https://figshare.com/articles/software/Studies_on_proton_range_monitoring_by_utilizing_13N_peak_for_proton_therapy_applications/16635697>/2. The program reads the input data obtained from the MC model with incident proton energy interval of 5 MeV. All data used in the fitting function were obtained from PHITS simulation package. The proposed fitting function can be used for data extraction. The incident energy range and increment chosen in present fitting function covers most widely reported proton irradiation facilities. It uses two different interpolation techniques to reconstruct data with incident proton energy interval of 0.1 MeV. The linear interpolation uses linear polynomial to generate new set of data points in between known sets of data as;

$$d_{offset}=\frac{d_{0}\left( E_{1}-E_{inp} \right)+d_{1}\left( E_{inp}-E_{0} \right)}{E_{1}-E_{0}} (A1)$$

where *d_offset_*, *d_0_* and *d_1_* are the new offset distance, offset distance of preceding and following points, respectively. Similarly, *E_inp_*, *E_0_* and *E_1_* are the user input incident proton energy, energy of preceding and following points, respectively. Considering the cubic spline interpolation, the value of function *f(x)* are calculated in *n* base points. Furthermore, the coefficient for the cubic spline interpolation (i.e., spline coefficients) are computed. Then spline interpolation would be performed based on the obtained coefficient for the set of base points. Since the polynomial used here is cubic, there will be three coefficients that needs to be determined that are; *b(i)*, *c(i)* and *d(i)* which *i* = 1,2,3,…,*n*. The following function shown in Eq. (A2) has been used in the present spline interpolation;

$$s\left( x \right)=y\left( i \right)+b\left( i \right)\times\left( x-x\left( i \right) \right)+c\left( i \right)\times\left( x-x\left( i \right) \right)^{2}+d\left( i \right)\times\left( x-x\left( i \right) \right)^{3} (A2)$$

The results of linear and non-linear cubic spline fitting of distance offset for various incident proton energies are shown in Fig. A1. From the results shown in Fig. A1, the linear and non-linear trend of fitting the Monte Carlo data can be clearly seen. Employing both of these interpolation techniques would help further increase the precision and accuracy of the estimated peak offset distances.

**Fig. A1. Linear and spline interpolation of Monte Carlo data for 13N and Bragg-peak offset distance.**

**
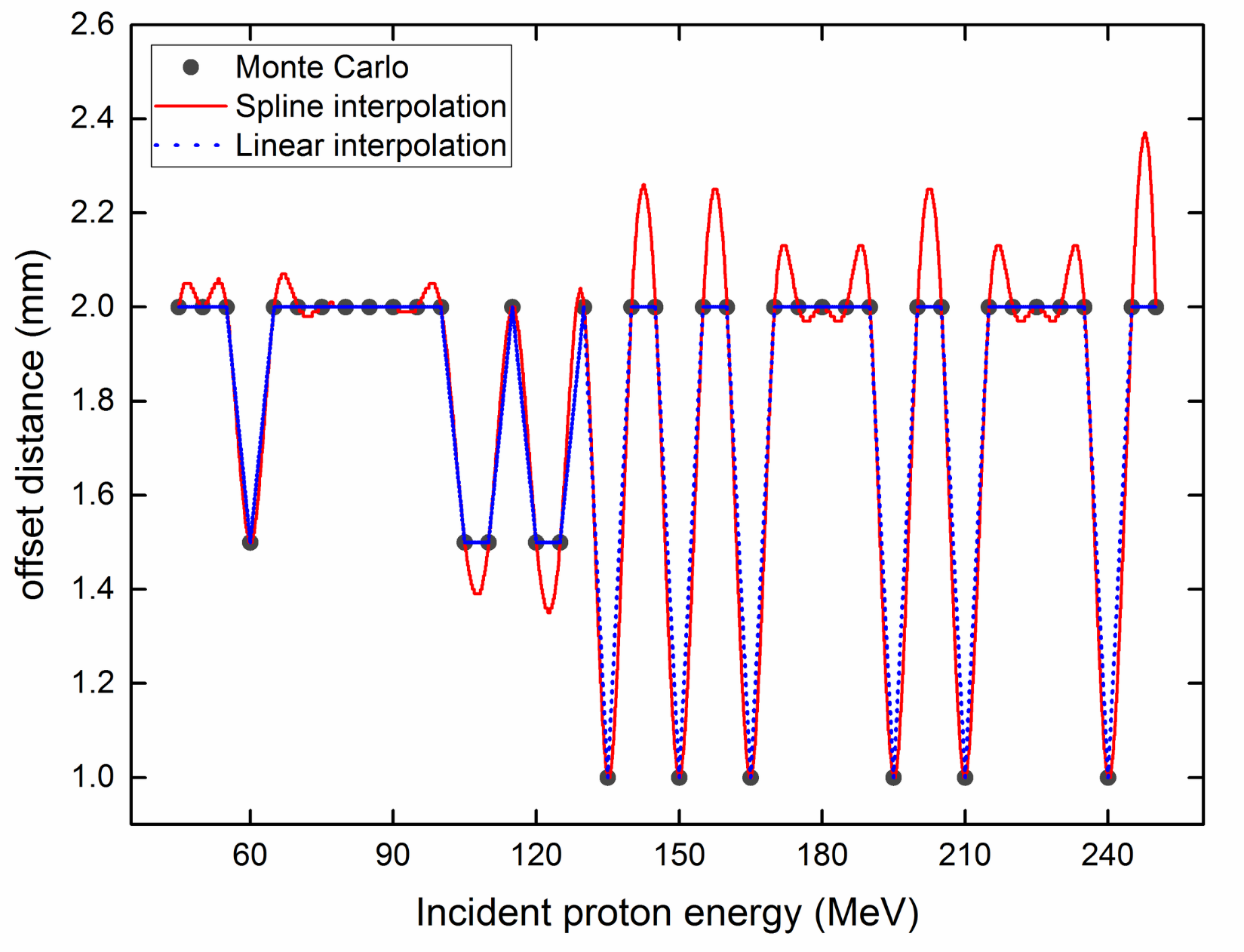
**
