# Supplementary material for: Proton range monitoring using 13N peak for proton therapy applications
Source: PLoS One. 2022 Feb 15;17(2):e0263521. doi: 10.1371/journal.pone.0263521 (PMC8846528; doi:10.1371/journal.pone.0263521)
Supplement: Attachment — Submitted filename: replies_20211228F.docx [file pone.0263521.s002.docx]

**Point-by-point summary of changes and replies according to the comments of Reviewers**

(Reviewer comments are in black; our replies in blue; changes applied to the manuscript are in green and underlined)

Please note that the line number presented in this reply letter corresponds to the version of the manuscript with Track Changes.

**Editor Comments:**

1. Please ensure that your manuscript meets PLOS ONE's style requirements, including those for file naming.

We thank the editor for the comment. We have now ensured that style requirements match those of PLOS ONE journal including those for file naming.

2. Thank you for stating the following financial disclosure:

a) Please clarify the sources of funding (financial or material support) for your study. List the grants or organizations that supported your study, including funding received from your institution.

We thank the editor for the comment. We have now amended the funding as follow;

This research work was funded by Agency for Medical Research and Development (AMED) under Grant Number 20he2202004h0402 and Ministry of Education, Culture, Sports, Science and Technology (MEXT) under Grant Number 21F21103.

We have now added the following to the cover letter.

This research work was funded by Agency for Medical Research and Development (AMED) under Grant Number 20he2202004h0402 and Ministry of Education, Culture, Sports, Science and Technology (MEXT) under Grant Number 21F21103.

b) State what role the funders took in the study. If the funders had no role in your study, please state: “The funders had no role in study design, data collection and analysis, decision to publish, or preparation of the manuscript.”

We thank the editor for the comment. The funders had no role in this study. We have now added the following sentence as recommended by the editor.

We have now added the following to the cover letter.

The funders had no role in study design, data collection and analysis, decision to publish, or preparation of the manuscript.

c) If any authors received a salary from any of your funders, please state which authors and which funders.

We thank the editor for the comment. None of the authors received salary from any of the funder stated in the present work.

None of the authors received salary from any of the funders mentioned in the present work.

d) If you did not receive any funding for this study, please state:
“The authors received no specific funding for this work.”
Please include your amended statements within your cover letter; we will change the online submission form on your behalf.

We thank the editor for the comment. We have now added the following changes to the revised cover letter, as recommended by the editor.

3. Thank you for stating the following in the Acknowledgments Section of your manuscript: [copy in statement]

We note that you have provided additional information within the Acknowledgements Section that is not currently declared in your Funding Statement. Please note that funding information should not appear in the Acknowledgments section or other areas of your manuscript. We will only publish funding information present in the Funding Statement section of the online submission form. Please remove any funding-related text from the manuscript and let us know how you would like to update your Funding Statement. Currently, your Funding Statement reads as follows:

The funders had no role in study design, data collection and analysis, decision to publish, or preparation of the manuscript.

Please include your amended statements within your cover letter; we will change the online submission form on your behalf.

We thank the editor for the comment. We have now removed any funding related sentence in the Acknowledgments Section of the revised manuscript.

We have added the funding information in the revised cover letter, as recommended by the editor.

4. We note that you have stated that you will provide repository information for your data at acceptance. Should your manuscript be accepted for publication, we will hold it until you provide the relevant accession numbers or DOIs necessary to access your data. If you wish to make changes to your Data Availability statement, please describe these changes in your cover letter and we will update your Data Availability statement to reflect the information you provide.

We thank the editor for the comment. We have used Figshare public repository to share our data. Figshare public repository has provided us the DOI number for ease of access. We have now included the DOI in the revised cover letter, as recommended by the editor.

All data and findings presented in this work can be downloaded from:

<https://doi.org/10.6084/m9.figshare.16635697.v2>

**We have now revised Figure 4, since the reference numbers were changed in the revised version.**

**We have included this revised figure in the cover letter as well.**

**Revised figure 4.**

**Reviewer #1**

1. The authors describe a method of utilizing the auto-activation of nitrogen-13 positron emitters to monitor the location of Bragg peaks in proton therapy. The work builds upon a paper by Cho et al. published in 2017. The novel contributions of the work are to 1) develop a N-13 peak - Bragg peak offset vs proton energy function and 2) develop a method of spectral analysis to determine the N-13 component of a PET voxel signal. With regard to contribution 1), I don’t believe a fitting function represents a significant contribution to the scientific community. Further, the precision of the data presented (whole integers) is on the same scale as the authors are attempting to fit to, which renders the fitting function obsolete.

We thank the reviewer for the comment. The approach chosen in previous work of Cho *et al*. is different from our proposed approach presented in this work. The authors mentioned the use of two data sets from fast and late PET scans, which would be subtracted from one another. The subtracted PET signal peaks and their offset was proposed to be used to perform proton range verification, without stressing specifically on the use of produced ^13^N positron emitting radionuclides. However, in present work, we mainly focused on the measured ^13^N radionuclide production to be used in proton range monitoring, which in fact provides useful insight to the physics of the problem and its potential applicability.

In addition, the previous investigation by Cho *et al*. was on investigating the possibility of using the emission from ^13^N radionuclide for estimating the Bragg-peak location. The authors employed Geant4 Monte Carlo package to verify and validate their results and test the proposed theory. From the previous work reported by Seravalli *et al*. [1], it was noted that no well-established theoretical model exists for such nuclear reactions (i.e., those producing positron emitters) and experimental data present in the current literature are in fact insufficient. Therefore, it would be pertinent to further verify these reported results using different Monte Carlo packages that use different physical models and data. Considering these, we believe the present work adds a valuable contribution to further verify and validate the previously reported results by employing PHITS Monte Carlo package.

With regard to contribution 1 as mentioned by the reviewer, we have simulated and obtained all the data from PHITS simulation package and the proposed fitting function was an addition for ease of data extraction. Certainly, the energy increment of 5 MeV considered in the proposed fitting function could be challenged, but it needs to be noted that there could be infinite number of incident energies, mainly from numerical point of view. The incident energy range and increment chosen in present fitting function covers most widely reported proton irradiation facilities.

1. Seravalli E, Robert C, Bauer J, Stichelbaut F, Kurz C, Smeets J, et al. Monte Carlo calculations of positron emitter yields in proton radiotherapy. Phys. Med. Biol. 2012; 57: 1659–1673.

We have now added the following to the lines 500-503 of the revised version of the manuscript.

All data used in the fitting function were obtained from PHITS simulation package. The proposed fitting function can be used for data extraction. The incident energy range and increment chosen in present fitting function covers most widely reported proton irradiation facilities.

2. With regard to contribution 2), the spectral analysis results appear promising, but no superior to the results presented by Cho et al. using an alternative method. Furthermore, as Cho et al. point out, N-13 peaks are only clearly present in monoenergetic Bragg peaks, which is of little practical use in proton therapy. Without performing the spectral analysis with spread-out Bragg peaks, the value of the work is questionable. Considering the points above, I cannot recommend the work for publication. If the authors are able to demonstrate the spectral analysis method is superior to that of Cho et al. when applied to spread out Bragg peaks, the work should be resubmitted for consideration.

We thank the reviewer for the comment. The spectral analysis employed in the present is indeed a promising approach. As mentioned in our reply to comment 1 of reviewer #1, we have employed a different approach in the present work when compared to the previous work of Cho *et al*. Therefore, the main focus of this work is to purely use emission from ^13^N radionuclide for proton range verification and energy validation of ^13^N production. In present paper, we have introduced our approach and in order to get an insight into the issue, we have considered monoenergetic proton beams; this way the underlying mechanism can be understood more clearly. We would like to emphasize that, the present work is not aimed at reporting superior results to that of Cho *et al*., it is rather introducing a different methodology and approach which would useful in advancing proton range monitoring. Regarding the ^13^N peak and its emission for different beam types, we aim to employ our introduced approach (which as mentioned differs from the approach reported by Cho *et al*.) for spread-out Bragg peaks in the future works. We strongly believe the current findings, developed tools and introduced approach would lay the foundation for future investigations.

We also would like to refer the reviewer to our replies to reviewer #2 and #3 and the main aim of our work and its clinical applications.

We have now added the following to the lines 395-397 of the revised version of the manuscript.

In future works, we aim to investigate the production of ^13^N and other positron emitting radionuclide by irradiating heterogenous phantoms with monoenergetic and spread-out Bragg-peak (SOBP) proton beams.

**Reviewer #2**

This is a very novel study by considering the generation of the positron emitters, especially the N13 peak proximal to the Bragg peak of proton therapy. The PHITS Monte Carlo simulation results show that the N13 peak is about 2 mm before the Bragg peak; therefore, the authors concluded that the N13 peak can be used to determine the Bragg peak location.

We thank the reviewer for the comment on our manuscript.

However, I think it is still very challenging to apply the method in this study to the actual clinical treatment cases to monitor the proton ranges. My concerns are listed below:

1. Can you elaborate the clinical significance of your study? For example, if it is a real patient being irradiated, is it practical to let the patient stay in the treatment couch waiting for 15 minutes to monitor the intensity of the positron emitters?

We thank the reviewer for the comment. Regarding the clinical significance of our study, it needs to be noted that intensity of the positron emitting radionuclides can be measured as long as they are being produced (i.e., when annihilation photon is emitted from the patient’s body). We refer the reviewer to the results shown in Fig. 3, for the distinct ^13^N signal measured immediately after proton irradiation. Another important point that needs to be considered is the fact that measuring annihilation photon provides mobility to the patient. The patient does not have to stick on the couch in the treatment room and measurements can be performed in another place. In addition, by measuring annihilation photons using PET or PEM system, relative time trend would be measured rather than absolute photon counts. Therefore, it would not be necessary to start the measurements immediately after proton irradiation. Having such flexibility would in fact be beneficial in realistic clinical treatment conditions.

Furthermore, the main reason behind the time dependent study from 15 to 75 minutes after irradiation is to show that ^13^N peak could still be detected. Certainly, very long waiting time may not be practical in realistic clinical treatment but in present work demonstrates the detection stability of ^13^N signal. Therefore, it should be noted that, there are no fixed waiting time constrains when applying this in realistic clinical conditions.

We have now added the following to the lines 312-319 of the revised version of the manuscript.

Regarding the clinical significance of our study, it needs to be noted that intensity of the positron emitting radionuclides can be measured as long as they are being produced (i.e., when annihilation photon is emitted from the patient’s body). Furthermore, measuring annihilation photon provides mobility of the patient. The patient does not have to stay on the treatment couch for the measurements. The measurements can be performed in another place. In addition, by measuring annihilation photons using PET system, relative time trend would be measured rather than absolute photon counts. Therefore, it would not be necessary to start the measurements immediately after proton irradiation. Having such flexibility would in fact be beneficial in realistic clinical treatment conditions.

2. The irradiated phantom is water-gel. I would say the reaction within the human tissues can be different from the water-gel. Have you considered the impact of the heterogeneities in human tissues on the N13 peak location?

We thank the reviewer for the comment. In present work, we have employed a homogeneous water-gel phantom mainly to eliminate any complex geometrical effect that might arise from the heterogeneities, such as those can be found in human tissue; this is to investigate the correlation between the production of ^13^N radionuclides and different incident proton energies. We aim to carry out a more extensive study on heterogenous phantoms (that closely resemble human tissues) in our future works.

We have now added the following to the lines 122-125 of the revised version of the manuscript.

In present work, we have employed a homogeneous water-gel phantom mainly to eliminate any complex geometrical effect that might arise from the heterogeneities, such as those can be found in human tissue; this is to investigate the correlation between the production of ^13^N radionuclides and different incident proton energies.

3. Only single energy beams have been considered in this study. Can you evaluate if this method is also applicable to using the SOBP, or even more complicated multi-field Intensity-modulated proton therapy cases?

We thank the reviewer for the comment. As per our reply to the previous comment (comment 2 of reviewer #2), in present work we mainly focused to extensively study the correlation between the production of ^13^N radionuclides and different incident proton energies. In addition, to eliminate any geometrical or incident beam complexities. Therefore, in current work we have employed single energy beam to investigate the ^13^N production and determine its offset distance with the Bragg-peak. We aim to carry out a more extensive study on heterogenous phantoms using SOBP beams in our future works.

(4) How will you accurately measure the spatial locations of these positron emitters? Using PET imagers around the patient during the proton therapy?

We thank the reviewer for the comment. The positron emitter could be precisely measured using PET (positron emission tomography) or PEM (positron emission mammography) systems around the patient after or during proton therapy. It is not necessary to perform PET or PEM measurements during or immediately after proton irradiations, please refer to our reply to comment 1.

We have now added the following to the lines 71-73 of the revised version of the manuscript.

The spatial locations of positron emitting radionuclides can be precisely measured using PET or PEM (positron emission mammography) systems around the patient after or during proton irradiation.

**Reviewer #3**

The paper contains interesting results in the reduction of the range uncertainties in proton therapy, and include useful tools developed that could help future research in this field, however, from my view, some parts of the work should be carefully reviewed before the publication.

We thank the reviewer for the comment.

**General comments**

1. In the Introduction to the work, various techniques used for the verification of the range in proton therapy collected in other works are summarized, however, references to previous papers from other authors based on the production of beta (-) emitters are not mentioned. From my perspective, these references have been used, but have not been properly referenced. In addition, it would be important to establish in a clear, explicit and summarized way, at the end of the Introduction, the main objective of the work and the methods followed.

We thank the reviewer for the comment. We have now revised the introduction and further elaborated on the relevant references, as recommend by the reviewer.

In addition, the main objective and methods followed were clearly summarized at the end of the introduction, as recommended by the reviewer.

The applicability of PET imaging in proton therapy monitoring was previously investigated by several groups [1-7]. In addition, the generated positron emitting radionuclides and their production channel was listed in previous studies; this was reported for proton interaction with human tissues [8].

1. Enghardt W, Debus J, Haberer T, Hasch BG, Hinz R, Jäkel O, et al. Positron emission tomography for quality assurance of cancer therapy with light ion beams. Nucl. Phys. A 1999;654: 1047c-1050c.

2. Parodi K, Enghardt W. Potential application of PET in quality assurance of proton therapy. Phys. Med. Biol. 2000;45: N151-N156.

3. Del Guerra A, Di Domenico G. Positron Emission Tomography as an aid to in vivo dosimetry for proton radiotherapy: a Monte Carlo simulation. TERA 1993, 93/10 TRA 9.

4. Oelfke U, Lam GK, Atkins MS. Proton dose monitoring with PET: quantitative studies in Lucite. Phys. Med. Biol. 1996;41: 177-196.

5. Parodi K, Enghardt W, Haberer T. In-beam PET measurements of β+ radioactivity induced by proton beams. Phys. Med. Biol. 2001;47: 21-36.

6. Paans AM, Schippers JM. Proton therapy in combination with PET as monitor: a feasibility study. IEEE Trans. Nucl. Sci. 1993;40: 1041-1044.

7. Dale Litzenberg. On-line Monitoring and PET Imaging of the Positron-Emitting Activity Created in Tissue by Proton Radiotherapy Beams. Ph.D. Thesis, University of Michigan. 1997. Available from: <https://deepblue.lib.umich.edu/handle/2027.42/130527>

8. Beebe-Wang J, Vaska P, Dilmanian FA, Peggs SG, Schlyer DJ. Simulation of proton therapy treatment verification via PET imaging of induced positron-emitters. IEEE Nucl. Sci. Symp. Conf. Record 2003;4: 2496–2500.

The overall objective of the present work is to investigate the effect of incident proton energy on the production of ^13^N positron emitting radionuclides, which in turn can be used to estimate the location of the Bragg-peak. The ^13^N and the Bragg-peak found to have an offset distance, and this was computed for wide range of incident proton energy. The current findings, developed tools and introduced approach would lay the pavement for future investigations and advancement in the field of proton range verification.

We have now added the following to the lines 63-66 of the revised version of the manuscript.

The applicability of PET imaging in proton therapy monitoring was previously investigated by several groups [1-7]. In addition, the generated positron emitting radionuclides and their production channel was listed in previous studies; this was reported for proton interaction with human tissues [8].

1. Enghardt W, Debus J, Haberer T, Hasch BG, Hinz R, Jäkel O, et al. Positron emission tomography for quality assurance of cancer therapy with light ion beams. Nucl. Phys. A 1999;654: 1047c-1050c.

2. Parodi K, Enghardt W. Potential application of PET in quality assurance of proton therapy. Phys. Med. Biol. 2000;45: N151-N156.

3. Del Guerra A, Di Domenico G. Positron Emission Tomography as an aid to in vivo dosimetry for proton radiotherapy: a Monte Carlo simulation. TERA 1993, 93/10 TRA 9.

4. Oelfke U, Lam GK, Atkins MS. Proton dose monitoring with PET: quantitative studies in Lucite. Phys. Med. Biol. 1996;41: 177-196.

5. Parodi K, Enghardt W, Haberer T. In-beam PET measurements of β+ radioactivity induced by proton beams. Phys. Med. Biol. 2001;47: 21-36.

6. Paans AM, Schippers JM. Proton therapy in combination with PET as monitor: a feasibility study. IEEE Trans. Nucl. Sci. 1993;40: 1041-1044.

7. Dale Litzenberg. On-line Monitoring and PET Imaging of the Positron-Emitting Activity Created in Tissue by Proton Radiotherapy Beams. Ph.D. Thesis, University of Michigan. 1997. Available from: <https://deepblue.lib.umich.edu/handle/2027.42/130527>.

8. Beebe-Wang J, Vaska P, Dilmanian FA, Peggs SG, Schlyer DJ. Simulation of proton therapy treatment verification via PET imaging of induced positron-emitters. IEEE Nucl. Sci. Symp. Conf. Record 2003;4: 2496–2500.

We have now added the following to the lines 83-87 of the revised version of the manuscript.

The overall objective of the present work is to investigate the effect of incident proton energy on the production of ^13^N positron emitting radionuclides, which in turn can be used to estimate the location of the Bragg-peak. The ^13^N and the Bragg-peak found to have an offset distance, and this was computed for wide range of incident proton energy. The current findings, developed tools and introduced approach would lay the pavement for future investigations and advancement in the field of proton range verification.

2. As an example of other works with beta (-) emitters used but no mentioned in this paper would be: Simulation of Proton Therapy Treatment Verification via PET imaging of Induced Positron-Emitters, (2003), J.J. Beebe-Wang et al. The Figure 4 in your paper is exactly the same as Figure 3 of this paper.

We thank the reviewer for the comment. We have now added the reference mentioned by the reviewer to the revived version of the manuscript.

The energy-dependent cross-section data for the ^16^O(p,2p2n)^13^N nuclear reaction reported by (1) ICRU 63 [28], (2) Del Guerra [29], and (3) Lizenberg [30], these were taken from Ref. [1] and are shown in Fig. 4.

1. Beebe-Wang J, Vaska P, Dilmanian FA, Peggs SG, Schlyer DJ. Simulation of proton therapy treatment verification via PET imaging of induced positron-emitters. IEEE Nucl. Sci. Symp. Conf. Record 2003;4: 2496–2500.

Please also see our reply to comment 1 of reviewer #3, which we have elaborated on more appropriate references.

We have now added the following to the line 278 and 282 of the revised version of the manuscript.

…these were taken from Ref. [1] and…

1. Beebe-Wang J, Vaska P, Dilmanian FA, Peggs SG, Schlyer DJ. Simulation of proton therapy treatment verification via PET imaging of induced positron-emitters. IEEE Nucl. Sci. Symp. Conf. Record,

2003;4: 2496–2500.

3. In Materials and Methods part, however, the work does not have a continuity, and references are constantly made to other previous works by the same authors. In this way, to understand and follow the paper, it would be necessary to consult up to seven additional papers, as collected by the authors. It is understandable to refer to works and methodologies developed in previous works, but the paper should contain the information and data necessary to be fully understood without having to consult so many additional references. The paper could be organized as a compendium of some of your previous work. However, this is not the main goal stated by you. As an example of this lack of continuity and permanent references to another works from the same authors would be:

3(a). Line 92 - More details regarding the MC simulation and modeling are available in our previous publications and the references therein [18-20].

We thank the reviewer for the comment. We have now added relevant explanations that would be useful to the present work by further elaborating the required information from our previous works, as recommended by the reviewer.

The Monte Carlo method is well-established in simulation of radiation transport. The stochasticity of interaction of radiation with matter can be conveniently considered using the Monte Carlo method; this is mainly accomplished by using pseudo random numbers at which determines the interaction with different nuclei and sampling the angular and energy distribution. Considering such stochasticity, the statistical analysis of the results would be important, in a way that low relative error in the estimated results would be desired.

We have now added the following to the lines 106-111 of the revised version of the manuscript.

The Monte Carlo method is well-established in simulation of radiation transport. The stochasticity of interaction of radiation with matter can be conveniently considered using the Monte Carlo method; this is mainly accomplished by using pseudo random numbers at which determines the interaction with different nuclei and sampling the angular and energy distribution. Considering such stochasticity, the statistical analysis of the results would be important, in a way that low relative error in the estimated results would be desired.

3(b). Line 110 - the obtained results were normalized to the primary incident proton (see Ref [2]).

We thank the reviewer for the comment. We have now added relevant explanations that would be useful to the present work by further elaborating the required information from our previous works, as recommended by the reviewer.

The tally results obtained from Monte Carlo simulations are mostly normalized per primary source particle by the Monte Carlo simulation package, as the absolute values would have no physical meaning. Therefore, the obtained results were normalized to the primary incident proton.

We have now added the following to the lines 135-138 of the revised version of the manuscript.

The tally results obtained from Monte Carlo simulations are mostly normalized per primary source particle by the Monte Carlo simulation package, as the absolute values would have no physical meaning. Therefore, the obtained results were normalized to the primary incident proton.

3(c). Line 135 - More details regarding compartmental modeling are available in our previous publications and our recently developed compartmental software [24-26].

We thank the reviewer for the comment. We have now added relevant explanations that would be useful to the present work by further elaborating the required information from our previous works, as recommended by the reviewer.

Compartmental modelling refers to the of modelling substance transport in a system consisting of multiple compartments (i.e., distinct regions/voxels), which is characterized by the transfer rates among the relevant compartments. The variations of certain substances or, more generally, the radionuclides in different compartments could be explained using sets of differential equations.

We have now added the following to the lines 163-167 of the revised version of the manuscript.

Compartmental modelling refers to the of modelling substance transport in a system consisting of multiple compartments (i.e., distinct regions/voxels), which is characterized by the transfer rates among the relevant compartments. The variations of certain substances or, more generally, the radionuclides in different compartments could be explained using sets of differential equations.

4. Finally, in Conclusions, considering that in some of previous works, the use of 13N to reduce the uncertainty of the range in proton therapy have been already discussed, and it was concluded that although the signal from that isotope can be characterized by Monte Carlo techniques, however, there are difficulties in real measurements due to its low intensity, it would be important that the authors justify this fact in this work, and the real usefulness of this technique could be established in a more obvious way.

We thank the reviewer for the comment. We agree with the reviewer. In realistic measurements using PET system, the measured signal could be weak and therefore it generates noisy images. There are various ways to circumvent the issue with weak signal and in turn denoise PET images. Recently, Guo *et al.* [1] introduced a novel kernel graph filtering method that could significantly tackle the issue with noisy PET images as a result of weak signal. The study performed by Guo *et al.* [1] was tested extensively using simulated and real life *in-vivo* dynamic PET datasets. The authors showed that the proposed method significantly outperforms the existing methods in sinogram denoising and image enhancement of dynamic PET at all count levels, and especially at low counts which measured signal from isotopes are weak. Therefore, the issue with weak signals that may create difficulties in realistic measurements could be solved rather effectively using denoising methods.

1. Guo S, Sheng Y, Chai L, Zhang J. Kernel graph filtering—A new method for dynamic sinogram denoising. PLoS ONE 2021;16: e0260374.

We have now added the following to the lines 191-199 of the revised version of the manuscript.

In realistic measurements using PET system, the measured signal could be weak and therefore it generates noisy images. There are various ways to circumvent the issue with weak signal and in turn denoise PET images. Recently, Guo *et al*. [1] introduced a novel kernel graph filtering method that could significantly tackle the issue with noisy PET images as a result of weak signal. The study performed by Guo *et al*. [1] was tested extensively using simulated and real life in-vivo dynamic PET datasets. The authors showed that the proposed method significantly outperforms the existing methods in sinogram denoising and image enhancement of dynamic PET at all count levels, and especially at low counts which measured signal from isotopes are weak. Therefore, the issue with weak signals that may create difficulties in realistic measurements could be solved rather effectively using denoising methods.

1. Guo S, Sheng Y, Chai L, Zhang J. Kernel graph filtering—A new method for dynamic sinogram denoising. PLoS ONE 2021;16: e0260374.

In addition, the total body PET scanner is another system that can be used to solve the issue with weak signals; this scanner has 200 cm axial field of view (FOV) and 40 times higher sensitivity than conventional PET systems [2].

2. Cherry SR, Jones T, Karp JS, Qi J, Moses WW, Badawi RD. Total-body PET: maximizing sensitivity to create new opportunities for clinical research and patient care. J. Nucl. Med. 2018;59: 3-12.

We have now added the following to the lines 199-201 of the revised version of the manuscript.

In addition, the total body PET scanner is another system that can be used to solve the issue with weak signals; this scanner has 200 cm axial field of view (FOV) and 40 times higher sensitivity than conventional PET systems [2].

2. Cherry SR, Jones T, Karp JS, Qi J, Moses WW, Badawi RD. Total-body PET: maximizing sensitivity to create new opportunities for clinical research and patient care. J. Nucl. Med. 2018;59: 3-12.

**Some detailed comments**

Line 3 – It is discovered. Sentence unclear. Did you discover that nuclear reaction?

We thank the reviewer for the comment.

We have now replaced “It is discovered that” by “Previously, it was reported that”.

We have now added the following to the line 3 of the revised version of the manuscript.

Previously, it was reported that…

Please rewrite Line 26 – Low exit dose. Sentence incorrect. Protons have not exit dose.

We thank the reviewer for the comment.

We have now changed “low exit dose” to “no exit dose”.

We have now added the following to the line 26 of the revised version of the manuscript.

…no exit dose…

Please rewrite Line 63 – Recently, it was reported the reaction. Sentence incorrect. The reaction and the threshold energy is well known and stated long time ago.

We thank the reviewer for the comment.

We have now changed “Recently, it was reported” to “It is well-known”.

We have now added the following to the line 66 of the revised version of the manuscript.

It is well-known…

Please rewrite. Line 98 - Table 1. Please justify why you choose this composition of the water-gel and why there is not Nitrogen in the phantom. In the same way, the percentage of Carbon is very high.

We thank the reviewer for the comment. The composition of water-gel phantom that was reported in previous investigations have rather very low nitrogen content [1,2]. Generally, some water-gel phantoms were produced experimentally by mixing agar powder (C_14_H_24_O_9_) with water (H_2_O), with the ratio of 1/100 (i.e., agar powder/water). Therefore, we have not considered nitrogen (^14^N) in the modelled water-gel.

1. Zhu X, España S, Daartz J, Liebsch N, Ouyang J, Paganetti H, et al. Monitoring proton radiation therapy with in-room PET imaging. Phys. Med. Biol. 2011; 56: 4041–4057.

2. España S, Zhu X, Daartz J, El Fakhri G, Bortfeld T, Paganetti H. The reliability of proton-nuclear interaction cross-section data to predict proton-induced PET images in proton therapy. Phys. Med. Biol. 2011;56: 2687–2698.

We have now added the following to the lines 97-101 of the revised version of the manuscript.

The composition of water-gel phantom that was reported in previous investigations have rather very low nitrogen content [1,2]. Generally, some water-gel phantoms were produced experimentally by mixing agar powder (C_14_H_24_O_9_) with water (H_2_O), with the ratio of 1/100 (i.e., agar powder/water). Therefore, we have not considered nitrogen (^14^N) in the modelled water-gel.

1. Zhu X, España S, Daartz J, Liebsch N, Ouyang J, Paganetti H, et al. Monitoring proton radiation therapy with in-room PET imaging. Phys. Med. Biol. 2011; 56: 4041–4057.

2. España S, Zhu X, Daartz J, El Fakhri G, Bortfeld T, Paganetti H. The reliability of proton-nuclear interaction cross-section data to predict proton-induced PET images in proton therapy. Phys. Med. Biol. 2011;56: 2687–2698.

Line 107 - Table 2. Please include the correct reference where you have picked these data.

We thank the reviewer for the comment. We have now added the appropriate reference for the data shown in Table 2.

…these data were taken from Ref. [1].

1. Beebe-Wang J, Vaska P, Dilmanian FA, Peggs SG, Schlyer DJ. Simulation of proton therapy treatment verification via PET imaging of induced positron-emitters. IEEE Nucl. Sci. Symp. Conf. Record 2003;4: 2496–2500.

We have now added the following to the lines 127-128 of the revised version of the manuscript.

…these data were taken from Ref. [1].

1. Beebe-Wang J, Vaska P, Dilmanian FA, Peggs SG, Schlyer DJ. Simulation of proton therapy treatment verification via PET imaging of induced positron-emitters. IEEE Nucl. Sci. Symp. Conf. Record 2003;4: 2496–2500.

Line 144 - M is the number of radionuclides produced. Sentence unclear. From my view, M represents the different types of radionuclides produced, numbered from j=1 to j=M. Please rewrite.

We thank the reviewer for the comment.

We have now changed “*M* is the number of radionuclides” to “*M* represents different types of radionuclides produced, numbered from *j*=1 to *j*=*M*”.

We have now added the following to the lines 175-176 of the revised version of the manuscript.

…*M* represents different types of radionuclides produced, numbered from *j=1* to *j=M*…

Line 193 - From SRIM and Continuous Slow Down Approximation for protons it would be possible to obtain the results of Table 3. Please justify more widely how these data was obtained with PHITS.

We thank the reviewer for the comment. SRIM uses CSDA model for computation of charged particle stopping power and in turn the range. The PHITS Monte Carlo package computes the average stopping power for the charged particles and nuclei either using the ATIMA package [1].

1. Geissel H, Scheidenberger C. Slowing down of relativistic heavy ions and new applications. Nucl. Instrum. Methods Phys. Res. B 1998;136: 114-124.

We have now added the following to the lines 234-235 of the revised version of the manuscript.

The PHITS Monte Carlo package computes the average stopping power for the charged particles and nuclei either using the ATIMA package [1].

1. Geissel H, Scheidenberger C. Slowing down of relativistic heavy ions and new applications. Nucl. Instrum. Methods Phys. Res. B 1998;136: 114-124.

Line 212 - My recommendation would be to include the average relative errors in the main paper, better than the caption of the Figure.

We thank the reviewer for the comment. We have now included the average relative errors in the main paper and removed these from figure caption.

We have now added the following to the lines 208-209 of the revised version of the manuscript.

The average relative errors were 0.169, 0.072, and 0.147 for energies 80, 160, and 240 MeV, respectively.

Line 237 – As already mentioned above, please include the correct reference.

We thank the reviewer for the comment. We have now included the correct reference, as recommended by the reviewer.

…these were taken from Ref. [1].

1. Beebe-Wang J, Vaska P, Dilmanian FA, Peggs SG, Schlyer DJ. Simulation of proton therapy treatment verification via PET imaging of induced positron-emitters. IEEE Nucl. Sci. Symp. Conf. Record 2003;4: 2496–2500.

We have now added the following to the line 282 of the revised version of the manuscript.

…these were taken from Ref. [1].

1. Beebe-Wang J, Vaska P, Dilmanian FA, Peggs SG, Schlyer DJ. Simulation of proton therapy treatment verification via PET imaging of induced positron-emitters. IEEE Nucl. Sci. Symp. Conf. Record 2003;4: 2496–2500.

Figure 1 - Please clarify in the paper that there is a gap of 25 cm of air from the proton beam to the phantom.

We thank the reviewer for the comment. We have now added the following to the revised version of the manuscript.

In the modelled irradiation setup, 25 cm of air gap was considered between the proton beam and the phantom.

We have now added the following to the lines 103-104 of the revised version of the manuscript.

In the modelled irradiation setup, 25 cm of air gap was considered between the proton beam and the phantom.

In conclusion, considering that the results could be interesting in the field of range monitoring for proton treatment applications, my advice to the authors would be they should take the time to justify better and more extensively these results, and that the writing of the paper must contain references in a proper and kind way.

We thank the reviewer for the comment. We have now implemented the reviewer comments in revised version of the manuscript. We sincerely appreciate all the reviewer comments and suggestions that significantly improved the quality of our manuscript.
